# Bevacizumab versus PARP-inhibitors in women with newly diagnosed ovarian cancer: a network meta-analysis

**DOI:** 10.1186/s12885-022-09455-x

**Published:** 2022-03-30

**Authors:** Young Ju Suh, Banghyun Lee, Kidong Kim, Yujin Jeong, Hwa Yeon Choi, Sung Ook Hwang, Yong Beom Kim

**Affiliations:** 1 Department of Biomedical Sciences, Inha University College of Medicine, Incheon, Republic of Korea; 2 Department of Obstetrics and Gynecology, Inha University hospital, Inha University College of Medicine, 27, Inhang-ro, Sinheung-dong, Jung-gu, Incheon, Republic of Korea; 3 Department of Obstetrics and Gynecology, Seoul National University Bundang Hospital, Seongnam-si, Gyeonggi-do Republic of Korea; 4 Department of Biostatistics, Korea University College of Medicine, Seoul, Republic of Korea

**Keywords:** Adverse events, Bevacizumab, *BRCA* mutation, Homologous recombination deficiency, Ovarian cancer, Poly(ADP-ribose) polymerase inhibitors, Progression-free survival

## Abstract

**Background:**

In women with newly diagnosed ovarian cancer, bevacizumab and poly (ADP-ribose) polymerase inhibitors (PARPi) exhibit improved progression-free survival (PFS) when administered concurrent with chemotherapy and/or maintenance therapy, but no study has directly compared their effects. Therefore, this study aimed to compare the efficacy and safety of bevacizumab and PARPi in women with newly diagnosed ovarian cancer using a network meta-analysis.

**Methods:**

PubMed, Medline, and Embase databases were searched, and five randomized trials assessing PFS in women with newly diagnosed ovarian cancer treated with either bevacizumab, PARPi, or placebo or no additional agent (controls) were identified. PFS was compared in the overall population with ovarian cancer, women with a *BRCA1/2* mutation (BRCAm) and women with homologous-recombination deficiency (HRD). Adverse events (grade ≥ 3) were compared in all populations of the included studies.

**Results:**

PARPi improved PFS significantly more than bevacizumab in women with a BRCAm (HR 0.47; 95% CI 0.36–0.60) and with HRD (HR 0.66; 95% CI 0.50–0.87). However, in the overall population with ovarian cancer, no significant difference in PFS was observed between women treated with PARPi and those treated with bevacizumab. PARPi exhibited the highest surface under the cumulative ranking probabilities value as the most effective treatment for PFS (PARPi vs. bevacizumab: 98% vs. 52% in the overall population with ovarian cancer; 100% vs. 50% in women with BRCAm; 100% vs. 50% in women with HRD). For adverse events, the risk of all treatments was similar. However, PARPi had a higher adverse risk than the control group (relative risk 2.14; 95% CI 1.40–3.26).

**Conclusions:**

In women with newly diagnosed ovarian cancer, PARPi might be more effective in terms of PFS compared to bevacizumab. The risk of serious adverse events was similar for PARPi and bevacizumab.

**Supplementary Information:**

The online version contains supplementary material available at 10.1186/s12885-022-09455-x.

## Introduction

Ovarian cancer is a common type of gynecologic cancer and the most common cause of death in women with gynecologic cancers [1]. Most women with ovarian cancer present with advanced-stage disease [2]. Although response rates are high for combined cytoreductive surgery and platinum-based chemotherapy, almost 80% of women develop recurrent disease [3].

Currently, targeted therapies are included in the standard first-line treatment of ovarian cancer. Vascular endothelial growth factor (VEGF) and angiogenesis have been shown to promote ovarian cancer progression, and bevacizumab, a humanized monoclonal antibody targeting VEGF-A, inhibits tumor angiogenesis [4]. In many studies, bevacizumab has improved survival of women with advanced and recurrent ovarian cancer [5–9]. *BRCA1/2* mutation (BRCAm) are a well-known cause of ovarian cancer and approximately 25% of ovarian cancers exhibit BRCAm [10]. Cancer cells harboring a BRCAm can be therapeutically targeted using poly (adenosine diphosphate [ADP]–ribose) polymerase inhibitors (PARPi), which prevent cancer cells from repairing chemotherapy-induced DNA damage [11, 12]. Many studies have reported survival benefits of PARPi in advanced and recurrent ovarian cancer [13–19].

Bevacizumab has been reported to improve progression-free survival (PFS) in women with newly diagnosed ovarian cancer when used concurrently with chemotherapy and subsequently as maintenance therapy [7, 8]. Recently, clinical studies have shown that PARPi maintenance therapy used after chemotherapy or concurrent chemotherapy improved PFS in a BRCAm cohort, a homologous recombination deficiency (HRD) cohort, and the overall population of women with newly diagnosed ovarian cancer [17–19].

Currently, bevacizumab, PARPi, or bevacizumab plus PARPi can be used to reduce recurrence after primary chemotherapy in women with newly diagnosed ovarian cancer that satisfy the eligibility criteria [20]. However, no study has directly compared the effects of bevacizumab and PARPi in this patient population. In the present study, we used a network meta-analysis approach to indirectly compare the effects of bevacizumab and PARPi on survival and adverse events in women with newly diagnosed ovarian cancer.

## Materials and methods

### Search strategy

We searched PubMed, Medline, and Embase databases in November 2021 for pertinent studies using combinations of the following keywords: (ovarian cancer OR tubal cancer OR peritoneal cancer) AND (bevacizumab OR niraparib OR rucaparib OR olaparib OR veliparib OR talazoparib) AND randomized trial (Additional file 1). Additional relevant studies not identified by database searches were identified by examining references provided by selected clinical studies and review articles.

### Selection criteria

The study inclusion criteria were studies of histologically diagnosed epithelial ovarian cancer (EOC), studies of newly diagnosed ovarian cancer, studies in which bevacizumab or PARPi was used, and randomized controlled studies. The exclusion criteria were non-case matched controlled studies, non-comparative studies, review articles, editorials, letters, abstracts, protocols, in vitro research studies, and irrelevant studies. To avoid including duplicate information, when studies included overlapping groups of patients, only the study with the most adequate data (including as many patients as possible) was included in the meta-analysis.

The process of study selection was based on the PRISMA 2020 statement [21].

### Data extraction and outcomes of interest

Two investigators independently extracted data of interest using a checklist. Any discrepancies between investigators were resolved by discussion. The eligible population of women with newly diagnosed ovarian cancer was classified into three groups based on whether they received bevacizumab, PARPi, placebo (the control group), or no additional agent (the control group). Data retrieved from studies were the name of the study, first author, year of publication, number of participants, numbers that received bevacizumab or PARPi or placebo or no additional agent, name of the PARPi administered, histologic type, number of disease progressions or deaths, number of women with a BRCAm, number of women with HRD, primary chemotherapy regimen, and number of adverse events (grade ≥ 3). Progression-free survival (PFS) was the principal outcome variable and was defined as the time between randomization and disease progression or death from any cause (in the absence of progression). PFS was analyzed in the following populations: the overall population with ovarian cancer, women with a BRCAm, and women with HRD. Adverse events (grade ≥ 3) in these treatment groups were compared in all populations of the included studies.

### Statistical analyses

Network meta-analysis was performed using a multivariate random effect model and a frequentist framework [22]. We investigated which treatment most effectively reduced the hazards of ovarian cancer progression (efficacy) and risks of adverse events (safety) by allowing multiple comparison treatment effects. Hazard ratios (HRs) were considered summary estimates of treatment response effect sizes for ovarian cancer progression, and relative risks (RRs) were considered summary estimates of effect sizes for adverse events. To determine whether a dispersion existed among HRs or RRs across studies, we used the I^2^ statistic and Cochran’s Q statistic, which are indexes of heterogeneity. Rank probabilities of treatments for efficacy and safety were estimated by surface under the cumulative ranking probabilities (SUCRA) [23]. When the treatment chosen is the best option, SUCRA values approach 1 (100%), while SUCRA for the worst treatment option approaches zero.

Statistical analysis was performed using R software (Version 4.1.1, ‘netmeta’ package; R Foundation for Statistical Computing, Vienna, Austria) and STATA software Version 14 (StataCorp LLC, College Station, Texas, USA). Ethical approval was not required because anonymous aggregate data were used.

## Results

### Search results and characteristics and assessments of risk bias

Our literature search initially identified 353 potentially relevant studies, and five randomized controlled studies that met the selection criteria were ultimately identified (Additional file 2). The characteristics of the included studies are provided in Table 1, and the results of our assessments of risk bias are provided in Additional file 3. The included studies enrolled 4657 women with newly diagnosed ovarian cancer (1389 from two studies on bevacizumab, 1129 from three studies on PARPi, and 2139 controls treated with placebo or chemotherapy alone) (Table 1). In the included studies, bevacizumab was used concurrently with chemotherapy and then as a maintenance therapy [7, 8]. PARPi was used as maintenance therapy after chemotherapy in two studies (olaparib and rucaparib) and used concurrently with chemotherapy and then as maintenance therapy in one study (veliparib) [17–19].

**Table 1:** Characteristics of the included studies in which women with newly diagnosed ovarian cancer underwent front-line chemotherapy

Authors	Design	Population	Number of participants	Treatment arms	PFS			Number of adverse events (grade ≥ 3)
					HR	95% CI	***P*** value	
Burger et al. (2011) [7], GOG 218	RCT, Phase 3	Overall population with ovarian cancer (Serous type: 85%, stage III: 74.5%, stage IV: 25.5%)	Bevacizumab: 625 Control: 623	*Bevacizumab:* (Carboplatin AUC6 + Paclitaxel 175 mg/m^2^) q21 × 6 cycles + Bevacizumab 15 mg/kg q21 for cycles 2 through 22 *Control:* (Carboplatin AUC6 + Paclitaxel 175 mg/m^2^ + Placebo) q21 × 6 cycles + Placebo maintenance * Bevacizumab or placebo was initiated at cycle 2, rather than cycle 1.	0.717	0.625–0.824	< 0.001	Bevacizumab: 408/607 Control: 356/608
Perren et al. (2011) [8], ICON 7	RCT, Phase 3	Overall population with ovarian cancer (Serous type: 69%, stage I, II: 18.4%, stage III: 68.4%, stage IV: 13.2%)	Bevacizumab: 764 Control: 764	*Bevacizumab:* (Carboplatin AUC5 or 6 + Paclitaxel 175 mg/m^2^) q21 × 6 cycles + Bevacizumab 7.5 mg/kg q21 concurrently for 5 or 6 cycles and continued for 12 additional cycles or until PD *Control:* (Carboplatin AUC5 or 6 + Paclitaxel 175 mg/m^2^) q21 × 6 cycles * Bevacizumab was omitted at cycle 1 if chemotherapy was started within 4 weeks of surgery	0.81	0.70–0.94	0.004	Bevacizumab: 491/745 Control: 419/753
Moore et al. (2018) [17], SOLO1	RCT, Phase 3	BRCAm cohort (High grade serous type: 96%, stage III: 83.1%, stage IV: 16.9%)	PARPi: 260 Control: 131	*Eligibility:* Women who had a complete or partial clinical response after platinum-based chemotherapy *Randomization:* After completion of platinum-based chemotherapy *PARPi:* Oral Olaparib 300 mg twice daily until PD *Control:* Placebo	0.3	0.23–0.41	< 0.001	PARPi: 208/260 Control: 42/130
González-Martín et al. (2019) [18], PRIMA	RCT, Phase 3	Overall population with ovarian cancer (Serous type: 95%, stage III: 64.9%, stage IV: 35.1%)	PARPi: 487 Control: 246	*Eligibility:* Women who had a complete or partial clinical response after platinum-based chemotherapy *Randomization:* Within 12 weeks after completion of the last dose of platinum-based chemotherapy *PARPi:* Oral Niraparib 300 mg once daily in 28-day cycles for 36 months or until PD (200 mg in some cases) *Control:* Placebo	0.62	0.5–0.76	< 0.001	PARPi: 341/484 Control: 46/244
		BRCAm cohort	PARPi: 152 Control: 71		0.4	0.27–0.62		
		HRD cohort (Serous type: 93.8%, stage III: 64.1%, stage IV: 35.9%)	PARPi: 247 Control: 126		0.43	0.31–0.59	< 0.001	
Coleman et al. (2019) [19], VELIA	RCT, Phase 3	Overall population with ovarian cancer (High grade serous type: 100%, stage III: 77.6%, stage IV: 22.4%)	PARPi: 382 Control: 375	*PARPi:* Carboplatin (AUC6, q21) + Paclitaxel (175 mg/m^2^ q21 or 80 mg/m^2^ q7) + oral Veliparib (150 mg twice daily) × 6 cycles followed by oral Veliparib 300 mg twice daily for 14 days and then oral Veliparib 400 mg twice daily until PD *Control:* Carboplatin (AUC6, q21) + Paclitaxel (175 mg/m^2^ q21 or 80 mg/m^2^ q7) + Placebo × 6 cycles + Placebo maintenance	0.68	0.56–0.83	< 0.001	PARPi: 332/377 Control: 285/371
		BRCAm cohort (stage III: 79.5%, stage IV: 20.5%)	PARPi: 108 Control: 90		0.44	0.28–0.68	< 0.001	
		HRD cohort (stage III: 77.7%, stage IV: 22.3%)	PARPi: 214 Control: 207		0.57	0.43–0.76	< 0.001	

*BRCAm BRCA1/2* mutations, *CI* Confidence interval, *HR* Hazard ratio, *HRD* Homologous-recombination deficiency, *PARPi* Poly (adenosine diphosphate [ADP]–ribose) polymerase inhibitor, *PFS* Progression-free survival, *PD* Progression of disease

### Indirect comparisons between PFS and adverse events (grade ≥ 3) after treatment with bevacizumab or PARPi

**Fig. 1:**
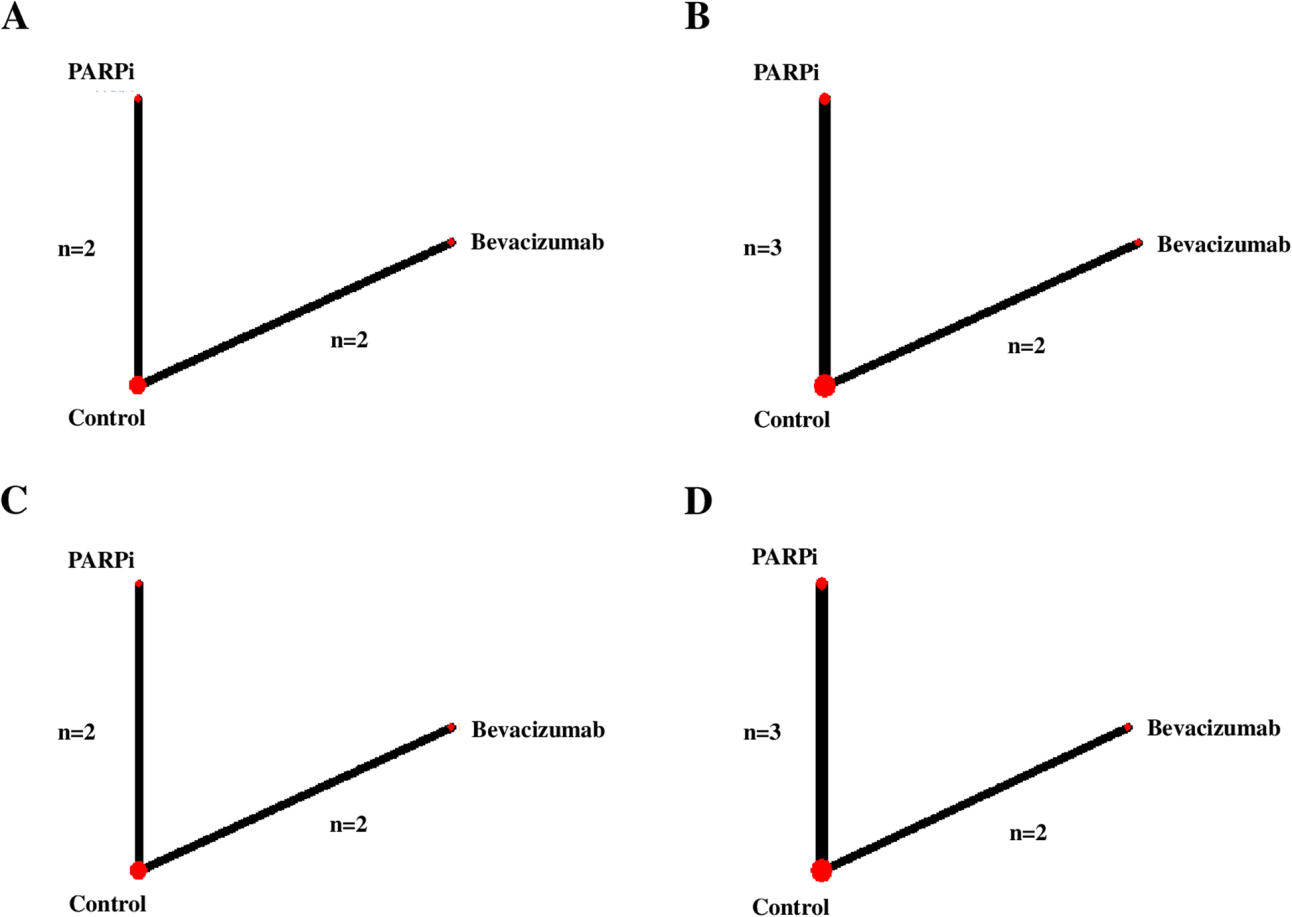
Network plots of treatments for PFS and adverse events. **A** PFS in the overall population with ovarian cancer, **B** PFS of women with a BRCAm, **C** PFS of women with HRD, and **D** Adverse events in all populations. The size of the three nodes (treatments) increased with the number of studies included in the corresponding nodes, and lines connecting two nodes were thickened with larger number of studies comparing the two treatments [24]. BRCAm, *BRCA1/2* mutation; HRD, homologous recombination deficiency

Figure 1 shows network plots of the pooled included studies on PFS in the overall population with ovarian cancer, women with a BRCAm, and women with HRD, and adverse events in all populations. Three treatment arms of bevacizumab, PARPi, and control treatment were identified in the plots. No significant heterogeneity was observed between studies for the comparison between bevacizumab and control treatments (I^2^ = 28.5%, *P* = 0.237 in PFS; I^2^ = 0%, *P* = 0.608 for adverse events) or between PARPi and control treatments (for PFS: I^2^ = 0%, *P* = 0.529 in the overall population with ovarian cancer; I^2^ = 20.3%, *P* = 0.285 in women with a BRCAm; I^2^ = 39.5%, *P* = 0.199 for women with HRD). However, the I^2^ for the PARPi vs. control comparison of adverse events was 98% (*P* < 0.001), indicating heterogeneity among studies.

**Fig. 2:**
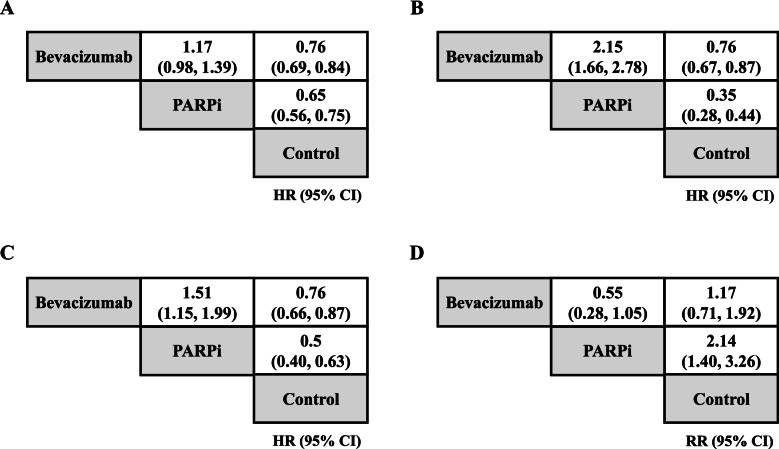
League tables of treatments for PFS and adverse events. **A** PFS in the overall population with ovarian cancer, **B** PFS for women with BRCAm, **C** PFS for women with HRD, **D** Adverse events in all populations. Hazard ratio (HR) or relative risk (RR) of the upper left treatment (intervention) vs. lower right (comparator) was estimated. BRCAm, *BRCA1/2* mutation; HRD, homologous recombination deficiency

**Fig. 3:**
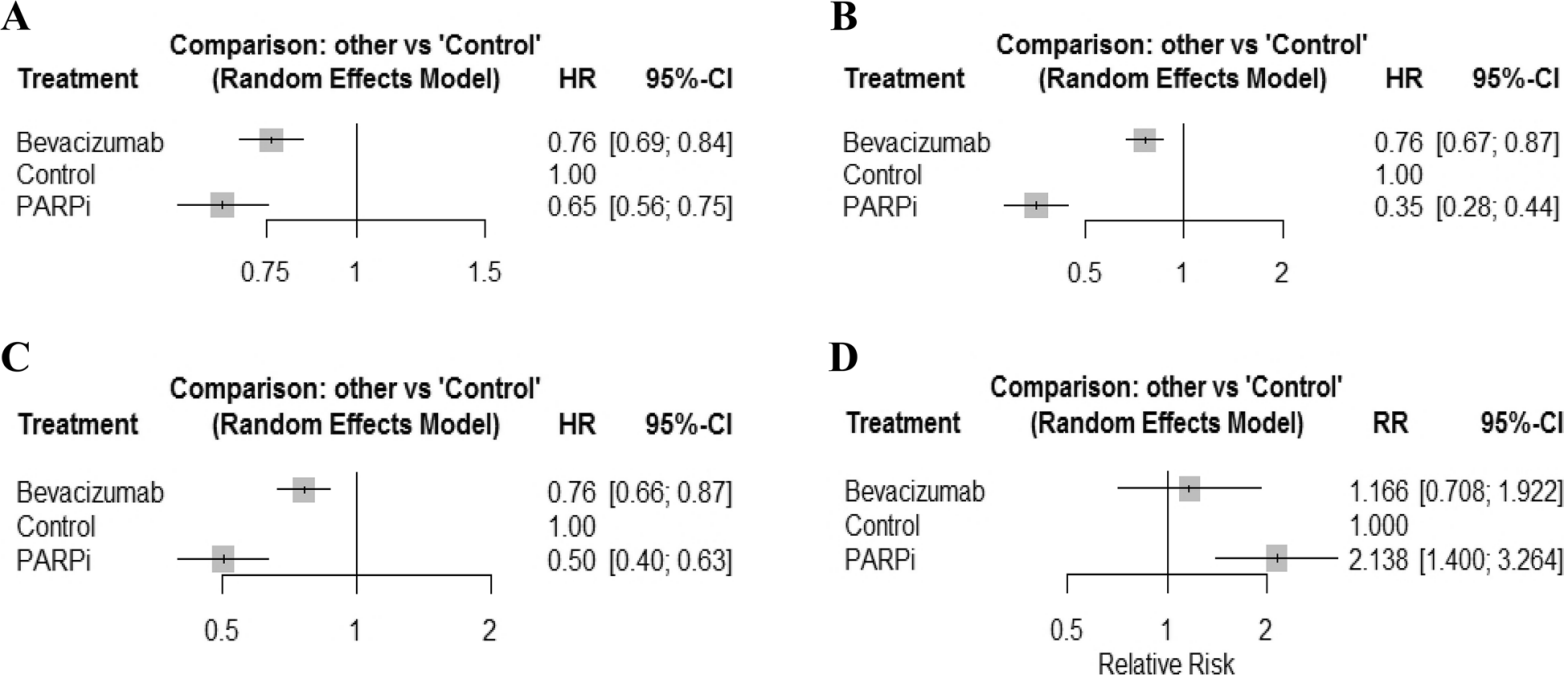
Forest plots of treatment for PFS and adverse events. **A** PFS in the overall population with ovarian cancer, **B** PFS for women with BRCAm, **C** PFS for women with HRD, **D** Adverse events in all populations. BRCAm, *BRCA1/2* mutation; CI, confidence interval; HR, hazard ratio; HRD, homologous recombination deficiency

Figure 2 presents the results of pairwise meta-analysis for PFS and adverse events. Bevacizumab exhibited lower hazards for ovarian cancer progression compared to the control treatments (HR 0.76, 95% CI 0.69–0.84 in the overall population with ovarian cancer; HR 0.76, 95% CI 0.67–0.87 for women with a BRCAm; HR 0.76, 95% CI 0.66–0.87 in women with HRD), and these results were significant. In addition, the hazard of ovarian cancer progression for PARPi was significantly lower than that of controls (HR 0.65, 95% CI 0.56–0.75 in the overall population with ovarian cancer; HR 0.35, 95% CI 0.28–0.44 for women with a BRCAm; HR 0.50, 95% CI 0.40–0.63 for women with HRD). For women with a BRCAm and women with HRD, the hazard of ovarian cancer progression for PARPi was significantly lower than that for those using bevacizumab (HR 0.47, 95% CI 0.36–0.60 for women with a BRCAm; HR 0.66, 95% CI 0.50–0.87 for women with HRD). However, in the overall population with ovarian cancer, no significant difference was observed between PFS achieved by PARPi or bevacizumab. For adverse events, with the exception of PARPi vs. control treatments, the risk of all treatments did not significantly differ. PARPi exhibited a higher risk for adverse events than did the control treatments (RR 2.14, 95% CI 1.40–3.26). Forest plots are presented in Fig. 3.

SUCRA curves for each treatment are shown in Table 2. In the overall population with ovarian cancer, women with a BRCAm, and women with HRD, PARPi had the highest SUCRA value, indicating it was a better treatment option for preventing ovarian cancer progression. For adverse events, control therapy had the highest SUCRA value.

**Table 2:** SUCRA values of treatments for PFS and adverse events

	Treatment efficacy		
	Treatment	SUCRA	Rank
PFS			
Overall population with ovarian cancer	PARPi	98%	1
	Bevacizumab	52%	2
	Control	0%	3
Women with a BRCAm	PARPi	100%	1
	Bevacizumab	50%	2
	Control	0%	3
Women with HRD	PARPi	100%	1
	Bevacizumab	50%	2
	Control	0%	3
Adverse events			
All populations	Control	93%	1
	Bevacizumab	57%	2
	PARPi	0%	3

*BRCAm BRCA1/2* mutation, *HRD* Homologous-recombination deficiency, *SUCRA* Surface under the cumulative ranking probabilities

## Discussion

It can be difficult to compare studies that have different designs, and head-to-head comparisons of the effects of therapeutic agents are particularly challenging. In such situations, some studies have performed indirect comparisons using a network meta-analysis [25, 26]. Here, we report the results of a study performed using this technique that indirectly compared the effects of bevacizumab and PARPi in women with newly diagnosed ovarian cancer. PARPi was found to improve PFS more than bevacizumab in women with a BRCAm and women with HRD. In the overall population with ovarian cancer, the effects of PARPi and bevacizumab on PFS were indistinguishable. However, SUCRA values demonstrated that PARPi had the highest probability of being the most effective treatment in terms of PFS in the overall population with ovarian cancer. On the other hand, all three treatment types were similar in terms of the risks of adverse events, with the exception that PARPi-containing treatments had a higher risk compared to control treatments.

In women with newly diagnosed ovarian cancer, both bevacizumab and PARPi improved PFS when administered concurrent with chemotherapy and/or maintenance therapy [7, 8, 17–19]. In two randomized studies, bevacizumab/platinum-based chemotherapy followed by bevacizumab maintenance therapy significantly improved PFS compared with platinum-based chemotherapy plus a placebo or platinum-based chemotherapy alone in the overall population with ovarian cancer [7, 8], Recently, PARPi significantly improved PFS compared with the placebo when used as maintenance therapy in two randomized studies performed in women with complete or partial clinical response to platinum-based chemotherapy [17, 18]. Moreover, in a randomized study, PARPi significantly improved PFS compared with platinum-based chemotherapy plus a placebo when administered concurrent with platinum-based chemotherapy and then as maintenance therapy [19]. Furthermore, these effects of PARPi have been reported in a BRCAm cohort, an HRD cohort, and the overall population with ovarian cancer [17–19]. Recently, one randomized study reported that, in women with newly diagnosed ovarian cancer, the addition of maintenance olaparib to bevacizumab/platinum-based chemotherapy significantly improved PFS without an increase in serious adverse events compared with bevacizumab/platinum-based chemotherapy in an HRD cohort (with or without a BRCAm) and a cohort with or without a BRCAm [27]. Therefore, it appears that several therapeutic strategies such as bevacizumab, PARPi, and bevacizumab plus PARPi can reduce the risk of recurrence after primary chemotherapy in women with newly diagnosed ovarian cancer. However, no study has directly compared the effects of bevacizumab and PARPi because of the different eligibility criteria and protocols used. Therefore, the agent that maximizes these therapeutic effects has yet to be determined. Based on our findings, we suggest PARPi to be the more effective therapeutic in terms of PFS in women with a BRCAm, women with HRD, and an overall population with ovarian cancer.

Adverse events can contribute to the choice between bevacizumab and PARPi. In randomized studies on bevacizumab, common adverse events (grade ≥ 3) were hypertension, thromboembolic events, neutropenia, and non-CNS bleeding [7, 8]. In randomized studies on PARPi, anemia, thrombocytopenia, neutropenia, fatigue, and nausea were common adverse events (grade ≥ 3) [17–19]. Our study showed that risks of adverse events (grade ≥ 3) did not vary for bevacizumab and PARPi.

In one recent network meta-analysis, PARPi improved PFS more than bevacizumab in women with platinum-sensitive recurrent ovarian cancer [25]. These findings were shown in an overall population with ovarian cancer, women with a BRCAm, and women with wild-type BRCA. In this prior network meta-analysis, an indirect comparison was performed of studies on bevacizumab that used bevacizumab/platinum-based chemotherapy followed by bevacizumab maintenance therapy, similar to our study. However, in contrast, studies on PARPi in that meta-analysis used only PARPi maintenance therapy after complete or partial response to platinum-based chemotherapy. Although there are differences, both this prior network meta-analysis and our study show that PARPi might be advantageous compared with bevacizumab in terms of PFS in women with platinum-sensitive recurrent ovarian cancer and women with newly diagnosed ovarian cancer.

The relevance of the present study stems from the comparison of effects of bevacizumab and PARPi in women with newly diagnosed ovarian cancer using network meta-analysis. To the best of our knowledge, this is the first study to compare the efficacy and safety of bevacizumab and PARPi in women with newly diagnosed ovarian cancer. However, the study has several limitations due to the different designs of the included studies. First, in two studies on bevacizumab and one study on PARPi, therapeutic agents were administered to women that received primary surgery for ovarian cancer, while in two studies, PARPi was administered to women with complete or partial clinical response to chemotherapy. Therefore, in the present study, all populations receiving bevacizumab and some populations administered PARPi included women with stable or progressive disease after surgery and who had started chemotherapy, indicating a bias toward better PFS for PARPi than bevacizumab. Second, in two studies on bevacizumab and one study using PARPi, these drugs were administered concurrently with chemotherapy and maintenance therapy, and in two studies, PARPi was administered as maintenance therapy. These concurrent therapies might have prolonged PFS because concurrent therapy was administered during the period used to measure PFS. Third, data in the overall population with ovarian cancer were used to analyze PFS in women with a BRCAm or HRD treated with bevacizumab because studies that used bevacizumab did not provide separate data on women with a BRCAm or HRD. Fourth, no randomized study directly compared the effects of bevacizumab and PARPi in women with newly diagnosed ovarian cancer. Therefore, this network meta-analysis provided an indirect comparison without analysis based on a combination of direct and indirect evidence.

## Conclusions

Although this study is limited by comparisons between studies with different designs, the indirect comparisons made using a network meta-analysis approach indicate that PARPi might be a more effective therapeutic strategy than bevacizumab with respect to PFS, and that the risk of serious adverse events posed by PARPi and bevacizumab are similar in women with newly diagnosed ovarian cancer. The results of this study provide valuable insights for selecting optimal front-line chemotherapy and maintenance therapy in women with ovarian cancer.

## Supplementary Information

**Additional file 1: Supplemental Table 1.** The search strategy used.

**Additional file 2: Supplemental Figure 1.** Flow chart of study selection.

**Additional file 3: Supplemental Table 2.** Assessments of risk of bias for the included studies.

## Data Availability

All data generated or analyzed during this study are included in this published article and its supplementary information files.

## Abbreviations

CI: Confidence intervals
HR: Hazard ratio
HRD: Homologous recombination deficiency
PARP: Poly (adenosine diphosphate [ADP]–ribose) polymerase
PARPi: Poly (adenosine diphosphate [ADP]–ribose) polymerase inhibitors
PFS: Progression-free survival
PD: Progression of disease
RR: Relative risk
