# Supplementary material for: Bevacizumab versus PARP-inhibitors in women with newly diagnosed ovarian cancer: a network meta-analysis
Source: BMC Cancer. 2022 Mar 30;22:346. doi: 10.1186/s12885-022-09455-x (PMC8969379; doi:10.1186/s12885-022-09455-x)
Supplement: Supplementary file 1 — Additional file 1: Supplemental Table 1. The search strategy used. [file 12885_2022_9455_MOESM1_ESM.docx]

**Additional file 1: Supplemental Table 1. The search strategy used.**

**Pubmed**

| # | **Search terms** |
| --- | --- |
| 1 | (ovarian cancer OR tubal cancer OR peritoneal cancer) AND (Bevacizumab OR Niraparib OR Rucaparib OR Olaparib OR Veliparib OR Talazoparib) AND randomized trial |

**Medline**

| **#** | **Search terms** |
| --- | --- |
| 1 | (ovarian cancer OR tubal cancer OR peritoneal cancer) AND (Bevacizumab OR Niraparib OR Rucaparib OR Olaparib OR Veliparib OR Talazoparib) AND randomized trial |

**Embase**

| **#** | **Search terms** |
| --- | --- |
| 1 | (ovarian cancer OR tubal cancer OR peritoneal cancer) AND (Bevacizumab OR Niraparib OR Rucaparib OR Olaparib OR Veliparib OR Talazoparib) AND randomized trial |

**Supplementary Figure S1.** Flow chart showing study selection.

**
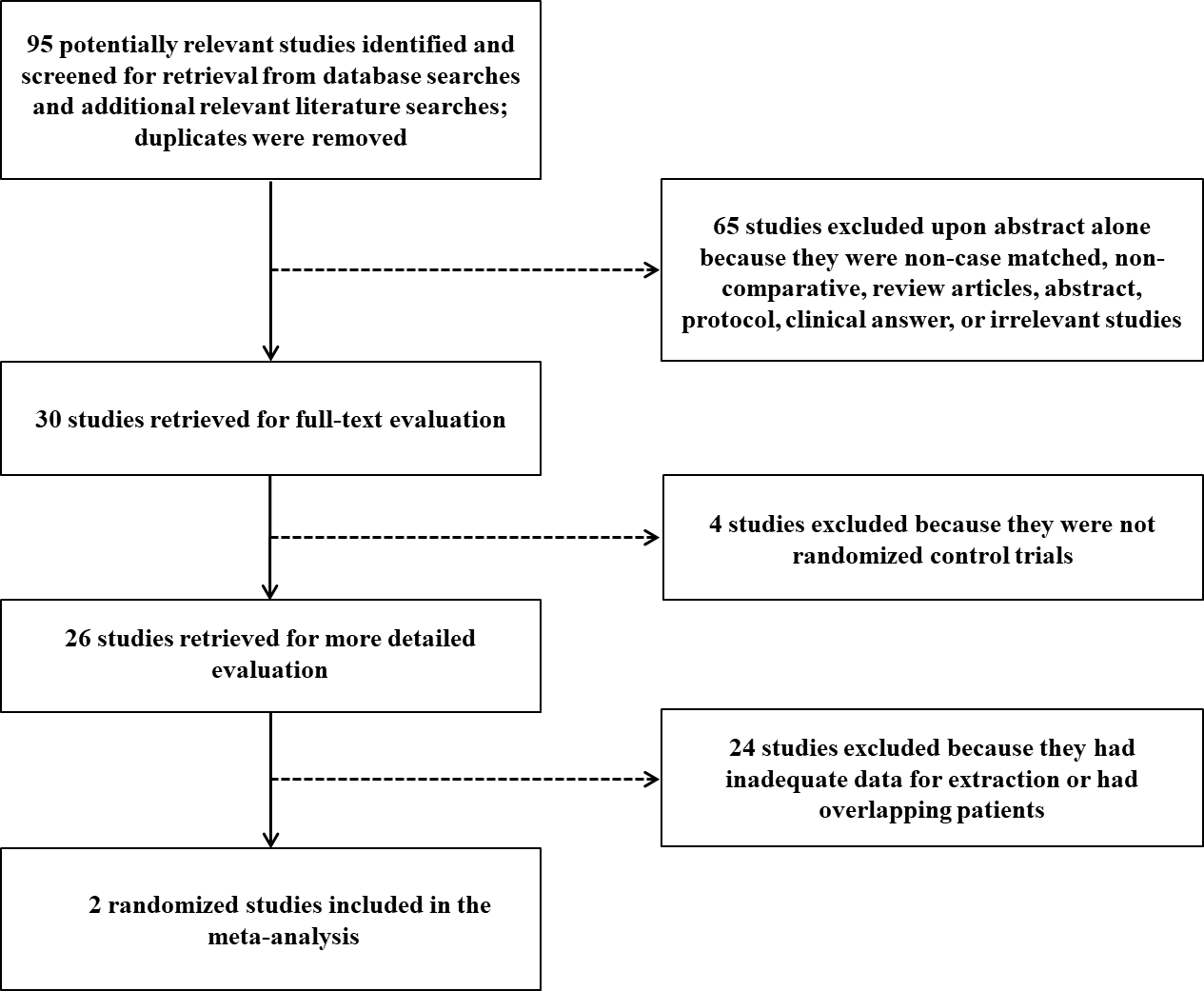
**

**Supplementary Figure S2.** Funnel plots: Progression-free survival between the germline and somatic BRCA1/2 mutation.
