# Supplementary figures and images for: Bevacizumab versus PARP-inhibitors in women with newly diagnosed ovarian cancer: a network meta-analysis
Source: BMC Cancer. 2022 Mar 30;22:346. doi: 10.1186/s12885-022-09455-x (PMC8969379; doi:10.1186/s12885-022-09455-x)

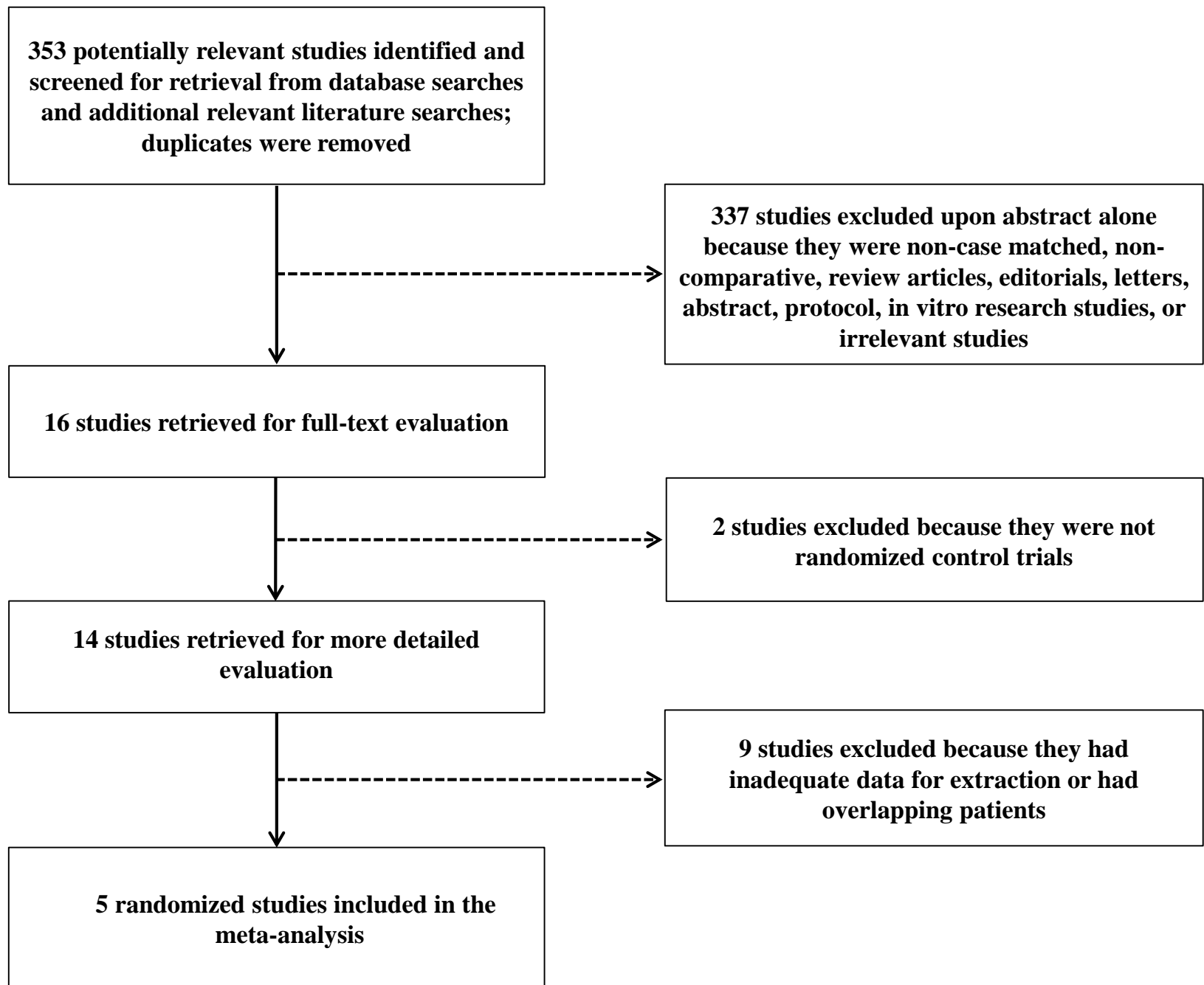

Supplement: Supplementary file 2 — Additional file 2: Supplemental Figure 1. Flow chart of study selection. [file 12885_2022_9455_MOESM2_ESM.pdf]
