# Supplementary material for: Bevacizumab versus PARP-inhibitors in women with newly diagnosed ovarian cancer: a network meta-analysis
Source: BMC Cancer. 2022 Mar 30;22:346. doi: 10.1186/s12885-022-09455-x (PMC8969379; doi:10.1186/s12885-022-09455-x)
Supplement: Supplementary file 3 — Additional file 3: Supplemental Table 2. Assessments of risk of bias for the included studies. [file 12885_2022_9455_MOESM3_ESM.docx]

**Additional file 3: Supplemental Table 2. Assessments of risk of bias for the included studies.**

| **Study** | **Random sequence generation**  **(selection bias)** | **Allocation concealment**  **(selection bias)** | **Blinding of the participants and personnel (performance bias)** | **Blinding of outcome assessment**  **(detection bias)** | **Incomplete outcome data (attrition bias)** | **Selective reporting**  **(reporting bias)** | **Other bias** |
| --- | --- | --- | --- | --- | --- | --- | --- |
| Burger et al. (2011) ^9^,  GOG 218 | | | | | | | |
| Authors’ judgement | Low risk | Low risk | Low risk | Low risk | Low risk | Low risk | Low risk |
| Support for judgement | Adequate method for randomization | Adequate allocation concealment | Blinding of the participants and personnel | Adequate blinding for outcome assessors | No incomplete outcome data | Report of all outcomes | No additional bias |
| Perren et al. (2011) ^10^,  ICON 7 | | | | | | | |
| Authors’ judgement | Low risk | Low risk | Unclear risk | Unclear risk | Low risk | Low risk | Low risk |
| Support for judgement | Adequate method for randomization | Adequate allocation concealment | Unclear blinding of the participants and personnel | Unclear blinding for outcome assessors | No incomplete outcome data | Report of all outcomes | No additional bias |
| Moore et al. (2018) ^11^,  SOLO1 |  |  |  |  |  |  |  |
| Authors’ judgement | Low risk | Low risk | Low risk | Low risk | Low risk | Low risk | Low risk |
| Support for judgement | Adequate method for randomization | Adequate allocation concealment | Blinding of the participants and personnel | Adequate blinding for outcome assessors | No incomplete outcome data | Report of all outcomes | No additional bias |
| González-Martín et al.  (2019) ^12^,  PRIMA |  |  |  |  |  |  |  |
| Authors’ judgement | Low risk | Low risk | Low risk | Low risk | Low risk | Low risk | Low risk |
| Support for judgement | Adequate method for randomization | Adequate allocation concealment | Blinding of the participants and personnel | Adequate blinding for outcome assessors | No incomplete outcome data | Report of all outcomes | No additional bias |
| Coleman et al. (2019) ^13^,  VELIA |  |  |  |  |  |  |  |
| Authors’ judgement | Low risk | Low risk | Low risk | Low risk | Low risk | Low risk | Low risk |
| Support for judgement | Adequate method for randomization | Adequate allocation concealment | Blinding of the participants and personnel | Adequate blinding for outcome assessors | No incomplete outcome data | Report of all outcomes | No additional bias |
